# Phylodynamics of avian influenza clade 2.2.1 H5N1 viruses in Egypt

**DOI:** 10.1186/s12985-016-0477-7

**Published:** 2016-03-22

**Authors:** Abdelsatar Arafa, Ihab El-Masry, Shereen Kholosy, Mohammed K. Hassan, Gwenaelle Dauphin, Juan Lubroth, Yilma J. Makonnen

**Affiliations:** Food and Agriculture Organization of the United Nations (FAO) - Emergency Center of Transboundary Animal Diseases (ECTAD), 11 Al Eslah El Zerai St, P.O. Box, 2223, Giza, Egypt; National Laboratory for Veterinary Quality Control on Poultry Production (NLQP), Animal Health Research Institute, P.O. Box, 264, Giza, Egypt; Food and Agriculture Organization of the United Nations (FAO), Viale delle Terme di Caracalla, 00153 Rome, Italy

**Keywords:** H5N1 HPAI, Poultry, Sequence and phylogenetic analysis, Virus evolution, Phylodynamic, Clade 2.2.1, Egypt

## Abstract

**Background:**

Highly pathogenic avian influenza (HPAI) viruses of the H5N1 subtype are widely distributed within poultry populations in Egypt and have caused multiple human infections. Linking the epidemiological and sequence data is important to understand the transmission, persistence and evolution of the virus. This work describes the phylogenetic dynamics of H5N1 based on molecular characterization of the hemagglutinin (HA) gene of isolates collected from February 2006 to May 2014.

**Methods:**

Full-length HA sequences of 368 H5N1 viruses were generated and were genetically analysed to study their genetic evolution. They were collected from different poultry species, production sectors, and geographic locations in Egypt. The Bayesian Markov Chain Monte Carlo (BMCMC) method was applied to estimate the evolutionary rates among different virus clusters; additionally, an analysis of selection pressures in the HA gene was performed using the Single Likelihood Ancestor Counting (SLAC) method.

**Results:**

The phylogenetic analysis of the H5 gene from 2006–14 indicated the presence of one virus introduction of the classic clade (2.2.1) from which two main subgroups were originated, the variant subgroup which was further subdivided into 2 sub-divisions (2.2.1.1 and 2.2.1.1a) and the endemic subgroup (2.2.1.2). The clade 2.2.1.2 showed a high evolution rate over a period of 6 years (6.9 × 10^−3^ sub/site/year) in comparison to the 2.2.1.1a variant cluster (7.2 × 10^−3^ over a period of 4 years). Those two clusters are under positive selection as they possess 5 distinct positively selected sites in the HA gene. The mutations at 120, 154, and 162 HA antigenic sites and the other two mutations (129∆, I151T) that occurred from 2009–14 were found to be stable in the 2.2.1.2 clade. Additionally, 13 groups of H5N1 HPAI viruses were identified based on their amino acid sequences at the cleavage site and “EKRRKKR” became the dominant pattern beginning in 2013.

**Conclusions:**

Continuous evolution of H5N1 HPAI viruses in Egypt has been observed in all poultry farming and production systems in almost all regions of the country. The wide circulation of the 2.2.1.2 clade carrying triple mutations (120, 129∆, I151T) associated with increased binding affinity to human receptors is an alarming finding of public health importance.

**Electronic supplementary material:**

The online version of this article (doi:10.1186/s12985-016-0477-7) contains supplementary material, which is available to authorized users.

## Background

Influenza-A viruses contain eight segments of single-strand RNA (ssRNA) and they are continuously evolving overtime. Point mutations can introduce small changes known as genetic drift which mainly occurs because the virus polymerase lacks the proofreading property. These changes are thought to be selected by pressures that force the virus to mutate. Highly pathogenic avian influenza viruses of the H5N1 subtype caused severe outbreaks in 1996/97 in southern China and Hong Kong [1]. In recent years, the H5N1 viruses spread from Asia to Europe and then to Africa, becoming endemic in poultry in parts of Asia and Egypt with frequent transmission to humans. In Egypt, the H5N1 HPAI virus (clade 2.2) was first reported in poultry in February 2006. Since then, the virus has spread rapidly among commercial and backyard flocks in most of the governorates [2]. Human infection rates were still rising as of June 2, 2014, reaching 175 infections with 63 deaths. By May 2015, infections reached 342 with 114 deaths due to the emergence of a new cluster originated from 2.2.1.2 [3]. The H5N1 viruses were isolated from ducks, chickens, and humans in Egyptian households and clustered into a distinct genetic group designated as 2.2.1. The majority of viruses derived from vaccinated poultry in commercial farms belonged to the 2.2.1.1 clade of variant viruses [4–6].

Genotypic characterization of avian influenza H5N1 viruses with the study of the evolutionary dynamics of circulating viruses will promote understanding of the virus evolution in a particular place. In a situation like Egypt, the genetic diversity of viruses leads to the production of heterogeneous genotypes [5]. However, the mechanisms associated with the genotype diversity of H5N1 viruses have still not been investigated [7]. Genetic phylogeny is currently considered the gold standard in characterizing viral genomics, transmission, and molecular evolution. Attempts to analytically trace the migration of viruses through evolutionary history have been done to infer migratory events [8]. In order to enhance the understanding of H5N1 HPAI virus epidemiology and the disease dynamics, particularly in endemic countries, regular linking of epidemiological data from individual outbreaks with the respective sequence information is of paramount importance to decide the activity of efficient disease control.

The aim of this work was to study the evolution of Egyptian H5N1 viruses from 2006 to 2014 using longitudinal epidemiological and virological data. The phylogenetic analysis of the HA gene linked with spatial data analysis can help us to understand the geographic spread of those viruses. This work aimed to describe the cluster dynamics of circulating viruses and evaluate the persistence of H5N1 strains in annual epidemics. Through that analysis, it was possible to determine the evolution rates of the HA gene and to characterise different H5N1genotypes in poultry in Egypt.

## Results and discussion

### Phylogenetic clusters

**Fig. 1:**
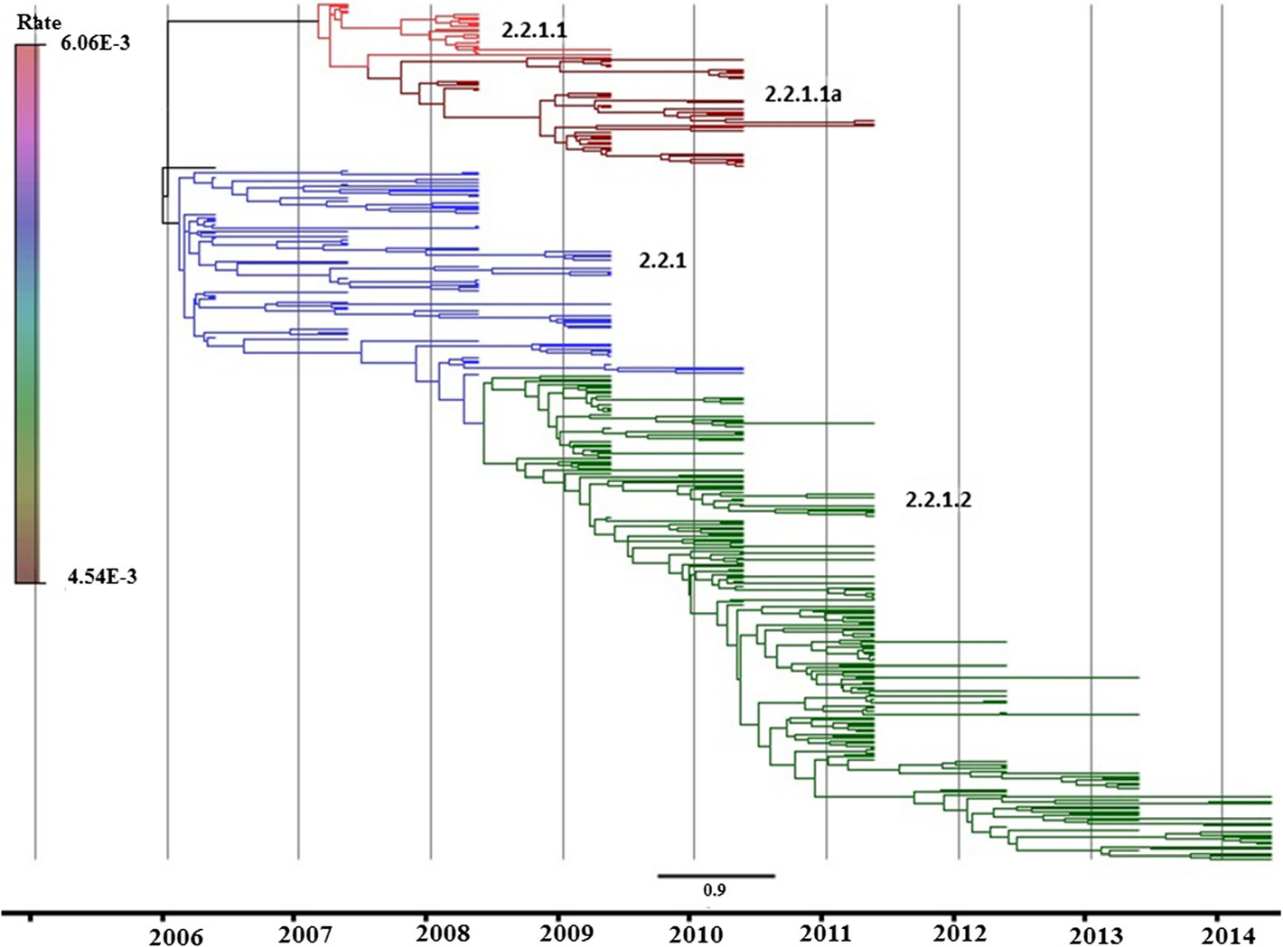
Phylogenetic analysis of H5N1 in Egypt during the period 2006–2014. The original viruses of clade 2.2.1 are marked in blue, while the cluster 2.2.1.2 clade which originated from 2.2.1 are marked in green. The variant clade 2.2.1.1 is divided into two clusters (2.2.1.1) marked in red and (2.2.1.1a) marked in pink. The genetic diversity has been illustrated on an annual scale

**Fig. 2:**
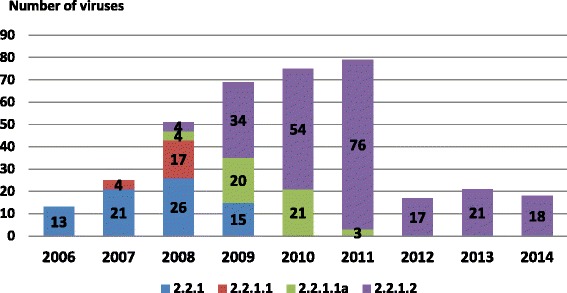
Distribution of the four H5N1 virus clusters in Egypt per year from 2006–2014 based on HA gene. The number of genetically characterized viruses does not reflect the number of recorded cases for each year

The phylogenetic analysis of the H5 gene of Egyptian viruses from 2006 to 2014 indicated the presence of two main subgroups—namely the classic 2.2.1 and the variant 2.2.1.1—according to the updated nomenclature of the WHO/OIE/FAO H5N1 Evolution Working group [9]. The classic group of clade 2.2.1 that was introduced into Egypt in 2006 remained stable through 2009 and represented the original viruses known at that time. The variant clade 2.2.1.1, which emerged in late 2007 from vaccinated commercial poultry, was subdivided into 2 clusters from 2008 to 2011 (2.2.1.1 and 2.2.1.1a). The first cluster emerged in late 2007 (2.2.1.1) and remained until 2009, while the second cluster (2.2.1.1a) emerged in 2008 and remained until 2011. Since then, these variant clusters have not been detected (Figs. 1 and 2). In 2008, the classic viruses evolved into a new clade 2.2.1.2 due to gradual accumulation of genetic mutations in the HA protein, and was the dominant cluster between 2009 and 2014 in both the household and commercial poultry sectors irrespective of their vaccination status (Figs. 1 and 2). Among the 368 H5 sequences analyzed in this study, there were 299 viruses of classic clade (75 belonged to 2.2.1, 224 to 2.2.1.2) and 69 viruses of variant clade (21 of 2.2.1.1 and 48 of 2.2.1.1a) (Fig. 2).

### Cluster dynamics

In general, the original viruses of clade 2.2.1 from 2006 to 2009 were distributed along populated areas of the Nile basin. They were mostly detected in the northern Delta region (53/75) with fewer cases (22/75) in Upper Egypt. The results from passive surveillance and notifications through the veterinary authority supported the same findings that Lower Egypt represented the highest record in comparison to Upper Egypt [10]. However, this finding requires further investigation because it may be due to a sampling bias, as most surveillance activity was directed to the Delta governorates at that time. 

**Fig. 3:**
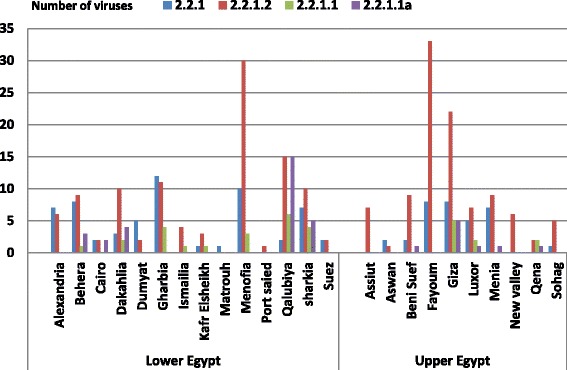
Distribution of the four clusters of genetically characterized H5N1 viruses collected from 2006 to 2014 per locality in Egypt

The endemic 2.2.1.2 cluster was identified first in 2008 and continues to circulate. It was widely distributed in most governorates in Egypt. Unlike 2.2.1, the 2.2.1.2 cluster was equally distributed between Lower and Upper Egypt (each with 112/224). The 2.2.1.2 viruses were mostly detected in Fayoum (37 virus) and Giza (24 virus) in Upper Egypt, as well as in Menofia (32 virus) in Lower Egypt, (Fig. 3).

From 2007 to 2011, most (51/69) viruses from the variant clade 2.2.1.1 were detected in the Delta area, with lower detection rates (18/54) in Upper Egypt (Giza, Beni suef, Menia, Luxor, and Qena governorates). The variant clade was highly prevalent (46/69) in commercial poultry farms, especially in Qalubiya (20/46), then Giza (6/46), Sharkia (5/46), and Dakahlya (5/46). Both 2.2.1.1 and 2.2.1.1a were first detected in Sharkia governorate with further expansion of both variant clusters to Qalubiya, Beheria, and Dakahlya in Lower Egypt and to Giza then to Upper Egypt (Additional file 1: Figure S1).

Analysis of virus population dynamics of the entire data set of the Egyptian H5N1 viruses showed a rise in genetic diversity in the 2.2.1.2 cluster from early 2008, shortly after the first introduction of the H5N1 viruses in the country in 2006. From 2009 to 2014, the 2.2.1.2 cluster exhibited a constant progressive adaptation to poultry and was considered to be an endemic cluster [11]. Genetically and antigenically distinct viruses emerged in Egypt in late 2007 after vaccination began in poultry (referred to as subgroups E and F [12] or subclade B [13] or clade 2.2.1.1 in this study) and estimated to have the highest divergence and rapid evolution rate.

**Fig. 4:**
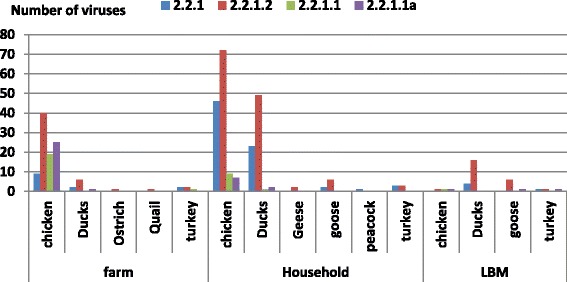
Distribution of the four clusters of genetically characterized H5N1 viruses collected from 2006 to 2014 per poultry species and farming sector in Egypt

The classic clade (2.2.1) and endemic clade (2.2.1.2) was widely distributed in the household poultry production sector involving chickens (118/134) and ducks (72/75). From 2009–2014, there was an increased detection of the 2.2.1.2 clade in live bird markets (LBM) (25/33), (Fig. 4). In this study, the variant clades (2.2.1.1 and 2.2.1.1a) were mainly reported in commercial chickens (44/93). Although most cases (38/69) were presented with unknown vaccination history, the variant clades were mainly reported in vaccinated flocks (27/69).

There is an apparent lack of disease notification and reporting in the commercial poultry sector in Egypt [14]. Thus HA gene sequences of H5N1 viruses since 2012 from this sector is lacking in most governorates. Phylogeography can highlight the drivers of H5N1 emergence and spread. Qalubiya appears to represent a popular location for virus transmission as also has been explored in previous study [15]. In addition, Sharkia and Dakahlia in Delta and Giza were of the same character as they have all virus clusters recorded in different time periods (Fig. 3). However, there remains uncertainty about virus spread to and from those locations and thus more research needs to be conducted in order to investigate this phenomenon.

### Analysis of selection pressures and evolutionary rates

The Nonsynonymous/Synonymous nucleotide substitution ratio (dN/dS) per site was greater than one for nine individual sites in the HA1 domain of the HA gene (Table 1), indicating the presence of positive selection driving the evolution of Egyptian H5N1 viruses in these sites. In particular, the 2.2.1.2 clade showed five distinct positively selected sites (120, 129, 154, 155 and 162), 2.2.1.1 has three prominent sites (140, 141 and 162), while 2.2.1.1a has five characteristic sites (140, 141, 154, 162 and 185) (Table 1). The results indicated that the 2.2.1.2 clade is under positive selection pressure that leads to more adaptation of the viruses to the environment and the maintenance of its endemic state.

**Table 1:** Positive selection pressure sites in the HA1 domain of the HA gene in different clusters of Egyptian H5N1 viruses

HA site	Mean ω (dN/dS)	Normalized ω (dN/dS)	Classic clade		Variant clade	
			2.2.1	2.2.1.2	2.2.1.1	2.2.1.1a
120	4.97	4.54	0	216	0	4
129	45.14	41.22	0	221	0	1
140	3.31	3.02	3	0	30	33
141	5.15	4.70	6	9	30	36
154	4.42	4.04	6	154	9	37
155	4.36	3.98	1	128	0	1
162	7.21	6.59	25	171	25	35
185	2.73	2.49	0	9	0	34
188	3.07	2.81	2	3	0	7

The estimates were made using the Single Likelihood Ancestor Counting (SLAC) method. dN/dS ratio of synonymous/ non synonymous per site. The number of viruses changed in each site. P value of < 0.05

The population dynamics analysis revealed a rapid increase in the genetic diversity of A/goose/Guangdong/1/96 lineage viruses from mid-1999 to early 2000 [7]. In this study, it was shown that the Egyptian H5N1 viruses exhibited high evolution dynamics in almost all governorates of the country. The viruses from clades 2.2.1.2 and 2.2.1.1a had the highest record of positive selection sites (Table 1), which may be attributed to vaccination pressure due to long-standing application of vaccines with high virus load in the endemic environment. This reflects the continuous adaptation of Egyptian viruses to the poultry and to their environment with persistent changes every season [16]. The genetic variation among the Egyptian viruses was previously reported and the presence of positive selection was recorded. In this regard, Cattoli et al. [13] indicated that evolutionary dynamics and positive selection significantly increased in virus populations in countries applying the avian influenza vaccination for H5N1, compared to viruses in countries that had never used vaccination. They also indicated that the rapid evolution of H5N1 viruses in Egypt was possibly linked to vaccination pressure due to sub-optimal use of vaccines.

**Fig. 5:**
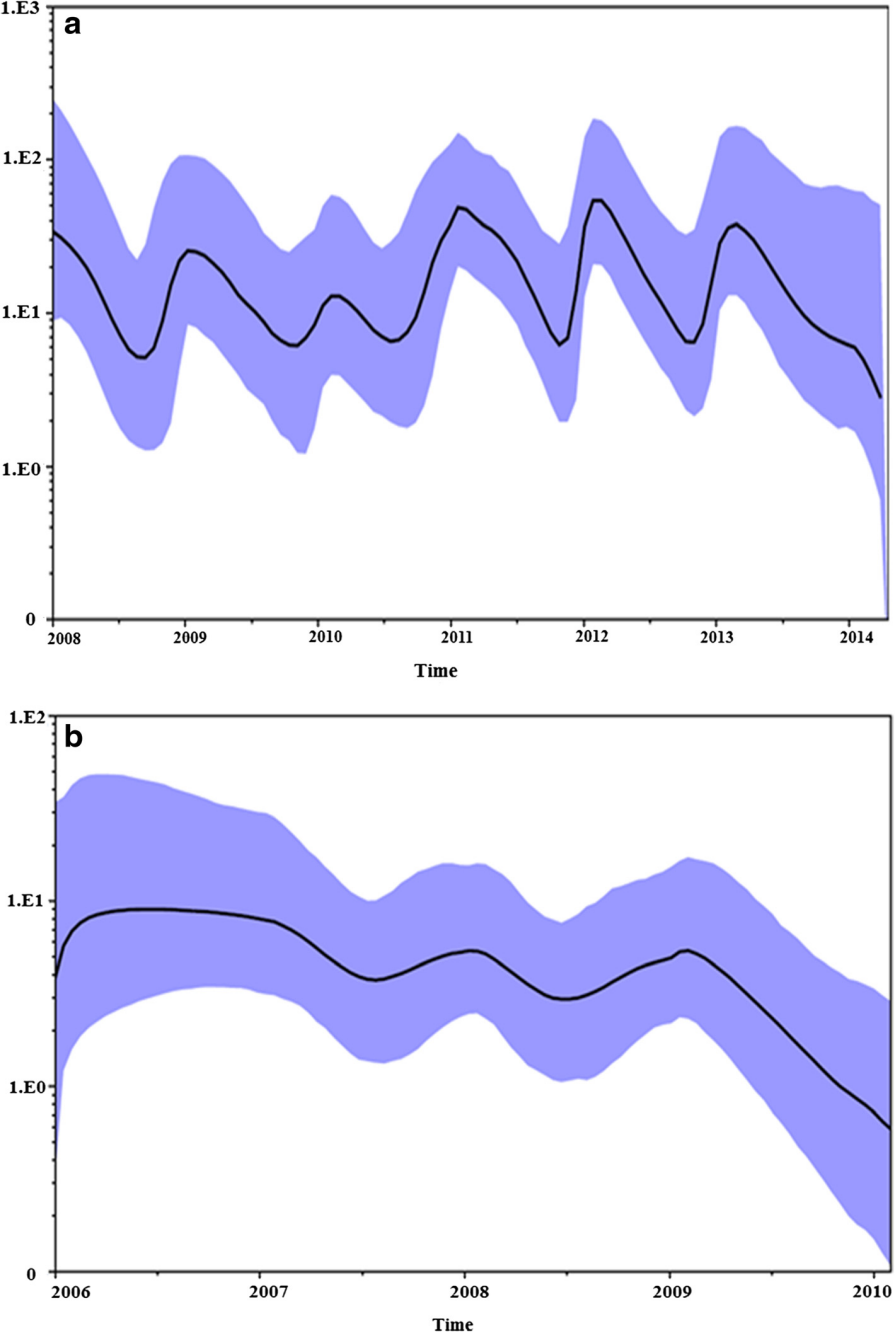
**a** The Bayseian skyride analysis of the 2.2.1.2 cluster from 2009 to 2014 showing changes in genetic diversity with 95 % HPD values. The rate of genetic diversity is proportional to increased annual prevalence peaks. **b** The Bayseian skyride analysis of the variant clusters from 2007 to 2011 showing changes in genetic diversity with 95 % HPD values. The genetic diversity showed a sharp decline in 2011

The Egyptian viruses showed a high rate of evolution since 2006, as the original clade 2.2.1 was 4 × 10^−3^ substitution/site/year and lasted for 4 years. The variant clade, conversely, had 6.1 × 10^−3^ substitution/site/year, distributed as 3.8 × 10^−3^ for 2.2.1.1 over 2 years and 7.2 × 10^−3^ for 2.2.1.1a over 4 years. The 2.2.1.2 clade showed higher and slower evolution rate in comparison to variant viruses, it was 6.9 × 10^−3^ over a period of 6 years (Table 2). In addition, the Bayesian skyride analysis of the 2.2.1.2 viruses from 2009 to 2014 showed that the genetic diversity is directly proportional to the annual prevalence peaks. The genetic diversity of the variant clusters from 2007 to 2010 showed a higher pattern of increase followed by a sharp decline in 2011(Fig. 5a, b).

**Table 2:** Evolutionary analysis of Egyptian H5N1 viruses

Virus cluster	Number of viruses	Duration (No. of years)	BEAST Mean (95% HPD)
Egypt classic 2.2.1	75	2006–2009 (4)	4 × 10^−3^ (3.4 − 4.7 × 10^−3^)
Egypt endemic 2.2.1.2	221	2009–2014 (6)	6.9 × 10^−3^ (1.3 − 9.6 × 10^−3^)
Egypt variant 2.2.1.1	31	2007–2008 (2)	3.8 × 10^−3^ (1.6 − 6.4 × 10^−3^)
Egypt variant 2.2.1.1a	38	2008–2011 (4)	7.2 × 10^−3^ (5.8 − 8.8 × 10^−3^)
Total Egypt variant 2.2.1.1	69	2007–2011 (5)	6.1 × 10^−3^ (4.8 − 7.3 × 10^−3^)
Total Egypt H5N1-2.2.1	365	2006–2014 (9)	5 × 10^−3^ (2.1 − 6.7 × 10^−3^)

Evolutionary rate subs/site/year (X10^−3^)

The evolutionary analysis of Egyptian viruses revealed that these viruses have progressive rates of evolution. The factors related to this increase were mainly attributed to the sub-optimal use of vaccines and long-lasting virus persistence in the environment leading to the endemic prevalence of 2.2.1.2 viruses over six successive years. Cattoli et al. [13] revealed that the two main Egyptian clades (designated as A and B) have co-circulated in domestic poultry since late 2007 and exhibited different profiles of positively selected codons and rates of nucleotide substitution. The mean evolutionary rate of clade 2.2.1 H5N1 viruses was estimated in their study as 4.07 × 10^−3^ nucleotide substitutions per site, per year whereas clade 2.2.1.1 viruses possessed a markedly higher substitution rate (8.87 × 10^−3^) and that reflected the high genetic diversity among Egyptian viruses.

### Molecular characterization and genetic analysis of HA gene

The analysis of the 368 HA genes enabled us to examine changes in the receptor binding site (RBS), antigenic sites (AS) and the cleavage site.

### Changes in the receptor binding site

The most characteristic change in the receptor binding site of the Egyptian viruses included in this study was the observation of one amino acid deletion at site 129 (129∆) that was not recorded in the ancestral strain (A/goose/Guangdong/1/96). This change was linked to the emergence of 2.2.1.2 clade in 2008. The viruses with 129∆ were found in the majority of human infections in Egypt in 2009 and have been found in all H5N1 human infections afterwards [17]. The presence of the 129∆ mutation may affect the binding of the virus to human receptors. Another important change in the receptor binding site was linked to the variant clades (2.2.1.1 and 2.2.1.1a), which showed S129L substitution. The substitution was more pronounced in the 2.2.1.1a cluster and was linked with another substitution (P74S) in most cases (28/35) (Table 3).

**Table 3:** Mutations in the receptor binding and antigenic sites of Egyptian H5N1 viruses from 2006-14

HA mutation	Years of detection	Virus cluster				Total
		2.2.1	2.2.1.2	2.2.1.1	2.2.1.1a	
changes in RBS						
129∆	2008–2014	-	221	-	1	222
changes in AS						
P74S	2007–2009	3	-	31	38	25
S120N	2007–2014	-	88	3	4	94
S120D	2010–2014	-	128	-	-	128
I151T	2008–2014	-	223	-	4	227
D154N	2007–2014	6	154	9	37	206
R162K	2007–2014	8	169	23	17	217
R162I	2006–2013	17	1	-	-	18
R162E	2008–2011	-	1	2	18	21
R140G + S141P	2007–2011	3	-	26	29	58
R140G + S141L	2009–2010	-	-	4	4	8
S141P	2008–2014	6	9	-	3	18
129L + P74S	2008–2010	-	-	7	28	35
129∆ + S120N + I151T	2009–2014	-	86	-	-	86^a^
129∆ + S120D + I151T	2010–2014	-	126	-	-	126^a^

*RBS* the receptor binding sites, *AS* the antigenic sites

^a^Triple mutation associated with increased binding affinity to human cell receptors

The calculation was based on a total number of 368 viruses where 2.2.1 = 75, 2.2.1.2 = 224, 2.2.1.1 = 31 and 2.2.1.1 a =38

The most recently isolated viruses (n = 56) that were collected between 2012 and 2014 were examined for HA gene mutations and belonged to the 2.2.1.2 clade in which three mutations (129∆, I151T, and S120(D,N)) were shown to be constant (Table 3).

The loss of HA154–156 glycosylation site was shown to enhance H5N1 virus binding to terminally α-2,6 sialic acid receptors and so increased the transmissibility to mammals [17–19]. The majority of the Egyptian 2.2.1 viruses lacked this site. There were few (6/75) 2.2.1 viruses that had the HA154–156 glycosylation site. Conversely, the majority of the Egyptian 2.2.1.2 viruses (154/224) had this site (Table 3).

The H5N1 viruses from Egypt displayed four characteristic mutations (D43N, S120(D,N), (S,L)129∆, and I151T). The results showed that 57 % of the HA sequenced genes showed a triple mutation (129∆, S120(D,N), and I151T) (Table 3). These triple mutations are characteristic in 2.2.1.2 clusters from different bird species such as chicken, duck, turkey, geese, ostrich, and quail; however, few of the 2.2.1.2 viruses did not carry them. The percentage of those viruses with the triple mutation reached 100 % from 2012 to 2014. Two mutations of those (129∆, I151T) had increased attachment and infectivity to the human lower respiratory tract, but not in the larynx [17]; that indicates an increasing possibility of human infections in Egypt.

The majority of variant viruses of clade 2.2.1.1 had no changes in the receptor binding site at position 129 (129∆) (Table 3), and they were not responsible for any human infection in Egypt, except for one case in early 2008 during the beginning of widespread prevalence of this cluster [20]. In addition, Perovic et al. [21] indicated the extensive evolution of Egyptian H5N1 HPAI virus towards human. They reported that all G2 viruses (referred to as 2.2.1.2 clade in our study) displayed four characteristic mutations (D43N, S120(D,N), (S,L)129∆ and I151T). The other mutations that are linked to increased affinity to human receptors like “S223N, D183G, E186G, Q192R, Q222L and G224S” were not found in the Egyptian H5N1 viruses.

### Changes in the antigenic sites

Amino acid substitutions at the antigenic sites can cause antigenic drift, possibly leading to vaccine escape as observed in the field. In comparison to the virus introduced in 2006, the characteristic changes that occurred in the antigenic sites of HA gene are very specific to each cluster (Table 3). There were 35/75 of 2.2.1 viruses that showed no antigenic changes in comparison to the parent Egyptian virus of 2006 and the precursor virus A/Bar-headed Goose/Qinghai/5/05. In addition, 26/75 of the viruses showed one antigenic change at either P74S or D154(E,N) or R162(I,K) or S141P sites. The remaining (14/75) of 2.2.1 viruses showed two to three changes. The most predominant antigenic change for those viruses was R162I which has been observed in 17 viruses. Approximately 60 % (132/224) of the viruses that belong to 2.2.1.2 cluster have four mutations that occurred at the same sites, i.e., S120(D,N), I151(T,L), D154(A,N) and R162K (Table 3). The Egyptian variant viruses (2.2.1.1 and 2.2.1.1a) carry characteristic mutations in the antigenic sites (at positions 74, 129, 140, 141, 154 and 162), while 2.2.1.2 cluster carries characteristic mutations at positions 120, 129, 151, 154, and 162; all these mutations can differentiate each cluster of Egyptian viruses from the others. Four characteristic mutations P74S, R162K, 140G and 141P were frequently detected together in the variant cluster 2.2.1.1 (17/31), whereas the variant cluster 2.2.1.1a had another four characteristic mutation that occurred together in sites P74S, R162(K,E), 141P and D154N (Table 3).

There have been 21 potential antigenic sites identified in the HA of H5N1 HPAI. It was shown that a single amino acid change in the HA of H5N1 HPAI virus can affect immune response and protection [22]. The recent Egyptian viruses from 2013 and 2014 carry four prominent mutations in the antigenic sites (at positions 120, 151, 154 and 162), all of which belong to the endemic clade 2.2.1.2 viruses; two of these mutations (154 and 162) were distinguishing the new viruses from the earlier 2008–09 viruses, which indicate limited changes in these sites in comparison to the earlier viruses of this cluster. However the recent viruses circulating during the last few years in Egypt from 2011 showed a clear separation from the ancestral viruses indicating a gradual evolution of those viruses.

The antigenic analysis of the earlier H5N1 variant strains in Egypt demonstrated antigenic variation [12, 23], which was driven by multiple mutations primarily occurring in the major antigenic sites at the globular head of HA [24]. Other studies showed that the classic clade 2.2.1 strains are antigenically related and cross-reactive to the ancestral Asian H5N1 strains, but demonstrated weak cross-reactivity with the Egyptian variant 2.2.1.1 strains [23, 25]. The majority of these mutations, alongside the other 19 amino acid mutations, were located within or adjacent to the receptor binding domain (RBD) in the HA1 that may affect the virus replication and transmission. There were six conserved mutations in previously reported antigenic sites (D43N, S120(N,D), S129∆, I151T, D154N and R162K) between the early 2006 strain and the endemic 2.2.1.2 cluster strains. It was shown that the D43N mutation resulted in antigenic drift between classic 2.2.1 and 2.2.1.2 clusters [26].

The results of the present study showed clear differences among the virus clusters in terms of the absence or presence of certain changes in the antigenic sites. For instance, the majority of 2.2.1.2 cluster had four changes occurring at the same time in the antigenic sites (S120(D,N), R162K, I151(T,L) and D145(A,N)), while the latter two sites were lacking in the 2.2.1.1 cluster. Ibrahim et al., 2013 [26] observed an antigenic variation between different H5N1 clusters, especially between the variant 2.2.1.1a and the endemic 2.2.1.2 cluster strains showing a significant antigenic drift. Some residues were located in different antigenic sites like 133S, 154D and 156A, 190L and 192Q and 71L. They also confirmed that the 2.2.1.1 cluster showed a broader reactivity to all strains that represent different H5N1 clusters circulating in Egypt, as it shared residues with all the strains in the major antigenic sites.

### Changes in the cleavage site

There were 13 groups of viruses identified based on the amino acid sequences at the cleavage site. Most of the H5N1 HPAI virus isolates belonging to 2.2.1 (42/75), 2.2.1.2 (105/224) and 2.2.1.1a (7/38) as well as all the viruses that belong to 2.2.1.1 cluster (31/31) have a common cleavage site of “**ERRRKKR**”, described as the consensus cleavage site for clade 2.2 viruses [27]. However, the pattern “**EKRRKKR**” became dominant from 2013 and replaced the previous pattern which disappeared after 2012 (Table 4). The pattern “**EGRRKKR**” of amino acid sequence was exclusively present in 2.2.1.1a cluster and represented the highest proportion (23/38) among the other six patterns in the same cluster, while the pattern “**ERRRKR**” was observed only in 30.6 % (23/75) of the viruses that belong to the 2.2.1 cluster (Table 4).

**Table 4:** The patterns of the HA cleavage site of H5N1 virus isolates (2006–14) genetically characterized in Egypt

Cleavage site AA pattern		Years of detection	Cluster				Total
			2.2.1	2.2.1.2	2.2.1.1	2.2.1.1a	
ERRRKR		2006–2007	23				23
EKRRKR		2006–2007	2				2
ERRRRKR		2007	1				1
ERRRKKR		2007–2012	42	109	31	7	192
EKRRKKR		2008–2014	1	110			111
DGRRKKR		2009				1	1
EGRRKKR		2009–2011				23	22
KSRRKKR		2009	6				6
ERKRKKR		2010		1			1
EGRRKKR		2010				5	5
EGRRRKKR		2010				2	2
DRRRKKR		2010		2			2
KKRRKKR		2013		1			1
Total	13		75	224	31	38	368

The currently dominant amino acid cleavage site pattern “PQG**EKRRKKR**/GLF” is closely associated with the mutation 129∆ at the receptor binding site, and mutations at the antigenic sites (S120D, I151T, D145N and R162K) which are known to increase the binding ability to human receptors. All these mutations characterize the dominant cluster (2.2.1.2) from 2011 onwards [26]. The substitution R325G was found at the cleavage site in Egyptian 2.2.1.1a viruses, while R325K characterized recent 2.2.1.2 viruses from 2011–2014. The R325G substitution was shown to significantly reduce pathogenicity without altering the transmission efficiency of H5N1 HPAI virus [28] and shows that non-adaptive mutations can play a role in virus evolution as the 2.2.1.1a cluster disappeared in Egypt since 2011.

The high diversity of the HA gene in relation to some governorates indicates active virus circulation in different locations. In this study, the hypervariability of the HA gene was noticed in relation to the geographical location (data not shown). Viruses from Qalubiya, Giza, Menufia and Dakahlya governorates had the highest number of heterogeneous amino acid sequences of the HA gene. In addition, unequal virus distribution was noticed among governorates and that favour virus persistence in an endemic area (Fig. 3). All the above mentioned results support the existence of genetic diversity of HA gene in Egypt with progressive virus evolution in a model of intermittent re-emerging H5N1 viruses to a clean areas located inside an endemic environment.

## Conclusions

Evolution of H5N1 HPAI viruses in Egypt continues to occur in all poultry farming and production systems and in almost all regions of the country. From 2006 to 2014, two clades have been detected, each subdivided into two genetic clusters (2.2.1, 2.2.1.1, 2.2.1.1a and 2.2.1.2). The 2.2.1.2 has been the dominant cluster circulating since 2011. It is possible that viruses within the variant clusters were less fit than the viruses of the classic clade 2.2.1, ultimately giving rise to a group of endemic clade 2.2.1.2 viruses. The wide circulation of the 2.2.1.2 cluster carrying mutations associated with increased binding affinity to human receptors is an alarming finding of public health importance. Continuous monitoring of the circulating viruses and sequencing of HA and other genes, in particular the NA gene, is important to better select viruses for vaccine studies and to understand the evolution of viruses over time. Regular data sharing among professionals in the animal and public health sectors will allow linking of epidemiological and sequence information and will provide a clear picture on the virus evolution.

## Methods

### Nucleotide sequencing of HA gene

The H5N1 HPAI virus isolates and field samples from 368 cases of H5N1 were collected in Egypt during the period from 2006 to 2014. They were collected from different localities in Lower and Upper Egypt, different bird species (chicken, duck, turkey, geese, quail and ostrich) and different poultry value chain nodes like households, commercial poultry farms and live bird markets.

The full length HA gene sequencing has been conducted, where the ribonucleic acids (RNAs) of virus isolates or samples were extracted using QiaAmp viral RNA extraction kit (Qiagen, Germany) according to the manufacturer’s instructions. A one-step RT-PCR was conducted on the extracted RNAs using specific primers for Matrix (M) and H5 genes [29, 30]. The PCR products were purified using a QiaAmp purification kit (Qiagen, Germany). The HA gene sequencing was done using a Bigdye Terminator Kit (version 3.1; Applied Biosystems, Foster City, CA) on a 3130 Genetic Analyzer (Applied Biosystems, Foster City, CA). The sequencing of the HA gene was conducted at NLQP and the data were regularly submitted to the GenBank and are available at the National Center for Biotechnology Information (NCBI) Influenza Virus Resource. Recently, new sequence data from 2012–2014 were added in the GenBank under accession numbers of KJ522707-KJ522745 and KP209286-KP209303.

### Phylodynamics of HA gene

After excluding the sequences from duplicate strains, 365 out of 368 full-length HA genes of Egyptian H5N1 viruses were used for this analysis. For the estimation of the rates of nucleotide substitution among H5N1 viruses from Egypt, the Bayesian Markov Chain Monte Carlo (BMCMC) method (as implemented in BEAST v1.4.7) was applied [31]. The Bayesian GMRF skyride coalescent tree model was used [32]. The uncorrelated lognormal relaxed (UCLD) clock [33] that allows evolutionary rates to vary along branches within lognormal distributions was used and Hasegawa-Kishino-Yano (HKY) substitution model with empirical base frequencies and gamma site heterogeneity model at 4 categories. Mean evolutionary rates and divergence times were calculated using Tracer V.1.5 [34]. The phylogenetic trees were visualized with FigTree v.1.1.2 [35].

### Analysis of selection pressures

The site-specific selection pressures for the HA gene of Egyptian H5N1 viruses was measured as the ratio of nonsynonymous (dN) to synonymous (dS) nucleotide substitutions per site (dN/dS) or omega (ω). Normalized dN-dS was estimated as the raw dN-dS divided by the total length of the tree measured in the number of expected substitutions per nucleotide per site. The estimates were made using the Single Likelihood Ancestor Counting (SLAC) method available at the Datamonkey online version of the Hy-Phy package [36]. This analysis used input Neighbor Joining (NJ) phylogenetic trees estimated according to the HKY model of nucleotide substitution. A site with ω > 1 is indicating positive selection. Statistical distributions were used to model the variation in ω among sites, allowing a subset of sites to have ω >1 while the rest of the sequence may be under purifying selection with ω < 1 with p-value of less than 0.05 [37].

### Genetic characterization of HA gene

In the present study, HA genes of 368 Egyptian H5N1 viruses were genetically characterized and studied for evidence of genetic mutations in different parts of the gene, including receptor binding, antigenic and cleavage sites. Multiple and pairwise sequence alignments were constructed using the Clustal-W algorithm of Bio-edit® software V.7.1.11 [38]. The mutations in the receptor binding and antigenic sites were tabulated against the virus clusters to explore their proportion among the sequenced HA genes and in order to identify the common antigenic differences between the virus clusters. The acquisition or loss of glycosylation sites of known importance in the HA gene was recorded. The amino acid sequences at the receptor binding, antigenic and cleavage sites were grouped and tabulated per cluster and year.

## Availability of supporting data

The sequences of HA gene were submitted to the public GenBank database under accession numbers from KJ522707 to KJ522745 and from KP209286 to KP209303.

## Additional file

Additional file 1: Figure S1. Schematic diagram showing the distribution and dynamic pattern of different H5N1 clusters on the Map of Egypt. (GIF 67 kb)

## Abbreviations

AS: antigenic sites
del: deletion
dN: nonsynonymous
dS: synonymous nucleotide substitution
ECTAD: Emergency Center of Transboundary Animal Diseases
FAO: Food and Agriculture Organization of the United Nations
HA: hemagglutinin
HKY: Hasegawa-Kishino-Yano
HPAI: highly pathogenic avian influenza
HPD: highest posterior density
LBM: live bird market, BMCMC, Bayesian Markov Chain Monte Carlo
NCBI: National Center for Biotechnology Information
NJ: neighbour joining
NLQP: National Laboratory for Veterinary Quality Control on Poultry Production
PCS: proteolytic cleavage site
RBD: receptor binding domain
RBS: receptor binding site
RNAs: ribonucleic acids
RT-PCR: reverse transcription polymerase chain reaction
SLAC: Single Likelihood Ancestor Counting
UCLD: uncorrelated lognormal relaxed clock
