# Supplementary figures and images for: Phylodynamics of avian influenza clade 2.2.1 H5N1 viruses in Egypt
Source: Virol J. 2016 Mar 22;13:49. doi: 10.1186/s12985-016-0477-7 (PMC4802640; doi:10.1186/s12985-016-0477-7)

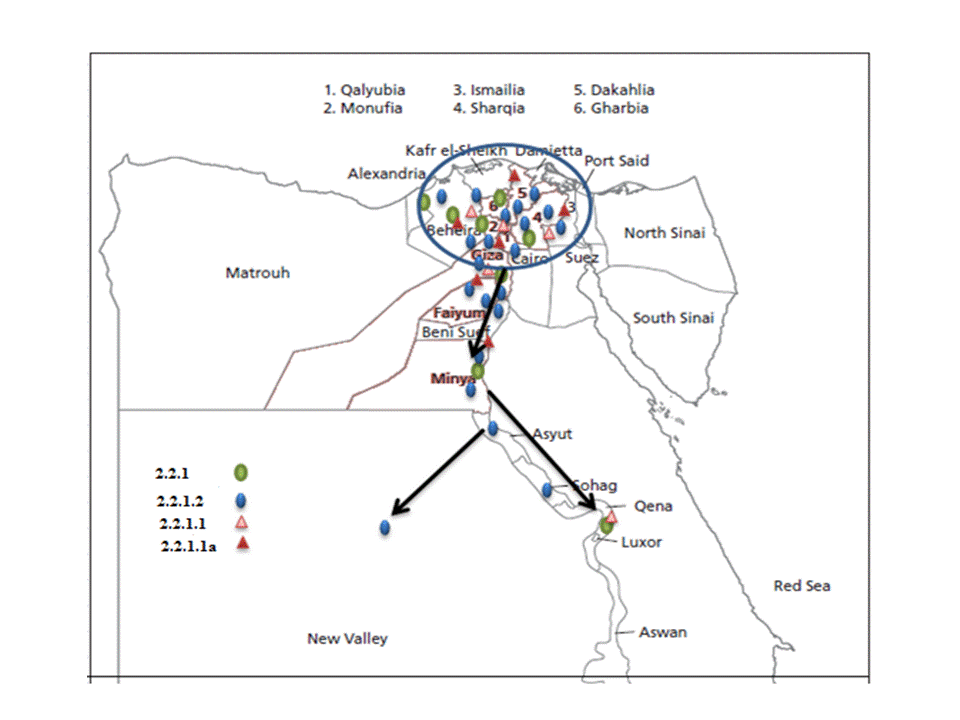

Supplement: Additional file 1: Figure S1. — Schematic diagram showing the distribution and dynamic pattern of different H5N1 clusters on the Map of Egypt. (GIF 67 kb) [file 12985_2016_477_MOESM1_ESM.gif]
